# Evidence for the existence of CD34^+^ angiogenic stem cells in human first‐trimester decidua and their therapeutic for ischaemic heart disease

**DOI:** 10.1111/jcmm.15800

**Published:** 2020-09-08

**Authors:** Long Bai, Lu Sun, Wei Chen, Kai‐Yu Liu, Chun‐Feng Zhang, Fei Wang, Gui‐Huan Zhang, Ye Huang, Jing‐Xuan Li, Ying Gao, Xin Sun, Wei Liu, Guo‐Qing Du, Ren‐Ke Li, Ming‐Li Huang, Hai Tian

**Affiliations:** ^1^ Department of Cardiovascular Surgery Second Affiliated Hospital of Harbin Medical University Harbin, Heilongjiang China; ^2^ Key Laboratory of Myocardial Ischemia Harbin Medical University Ministry of Education Harbin, Heilongjiang China; ^3^ Future Medical Laboratory Second Affiliated Hospital of Harbin Medical University Harbin, Heilongjiang China; ^4^ Department of Ultrasound Second Affiliated Hospital of Harbin Medical University Harbin, Heilongjiang China; ^5^ Department of Gynecology and Obstetrics First Affiliated Hospital of Harbin Medical University Harbin, Heilongjiang China; ^6^ Toronto General Hospital Research Institute University Health Network Toronto Ontario Canada; ^7^ Department of Surgery University of Toronto Toronto Ontario Canada

**Keywords:** angiogenic stem cells, bone marrow mesenchymal stem cells, cell transplantation, decidual CD34^+^ cells, ischaemic heart disease

## Abstract

Stem cell transplantation is nearly available for clinical application in the treatment of ischaemic heart disease (IHD), where it may be joined traditional methods (intervention and surgery). The angiogenic ability of seed cells is essential for this applicability. The aim of this study was to reveal the presence of CD34^+^ angiogenic stem cells in human decidua at the first trimester and to use their strong angiogenic capacity in the treatment of IHD. In vitro, human decidual CD34^+^ (dCD34^+^) cells from the first trimester have strong proliferation and clonality abilities. After ruling out the possibility that they were vascular endothelial cells and mesenchymal stem cells (MSCs), dCD34^+^ cells were found to be able to form tube structures after differentiation. Their angiogenic capacity was obviously superior to that of bone marrow mesenchymal stem cells (BMSCs). At the same time, these cells had immunogenicity similar to that of BMSCs. Following induction of myocardial infarction (MI) in adult rats, infarct size decreased and cardiac function was significantly enhanced after dCD34^+^ cell transplantation. The survival rate of cells increased, and more neovasculature was found following dCD34^+^ cell transplantation. Therefore, this study confirms the existence of CD34^+^ stem cells with strong angiogenic ability in human decidua from the first trimester, which can provide a new option for cell‐based therapies for ischaemic diseases, especially IHD.

## 1 INTRODUCTION

Stem cells have strong proliferation and differentiation abilities, among which the ability to differentiate to form vessels is critical to blood supply in MI.^1^, ^2^, ^3^ BMSCs, a kind of widely used seed cell, have been used as a satisfactory therapy for preclinical ischaemia animal models.^4^, ^5^ However, the effectiveness of some clinical trials is still controversial.^6^, ^7^ Therefore, the purpose of this research was to identify new stem cells that have strong angiogenic ability and low immunogenicity that could function as optimal seed cells for treating IHD.

Human decidua is a highly specialized tissue formed by the proliferation and redifferentiation of the endometrial stroma during pregnancy.^8^ The process of decidualization is accompanied by adaptive changes in numerous vascular structures, which enable embryo implantation and placentation.^9^ Thus, there may be angiogenic stem cells involved in the decidua. In addition, the decidua, which originates from a maternal source, is considered a physiologic barrier at the human maternal‐foetal interface that prevents immune rejection of the foetus.^10^ This indicates that it may have low immunogenicity.

CD34 is predominantly considered to be a marker of hematopoietic stem cells and vascular progenitor cells.^11^, ^12^, ^13^ Some experts have found that human umbilical cord blood‐derived CD34^+^ cells can repair the vascular structure of the retina and myocardium in mice.^14^, ^15^ This shows that CD34 is closely related to the formation of vessels. Other studies have confirmed that CD34^+^ cells of the mouse uterus are hemangioblasts, which have strong angiogenic ability.^16^ Therefore, we speculate that the human dCD34^+^ cell population from the first trimester may be made of angiogenic stem cells.

In our study, dCD34^+^ cells were isolated from healthy female decidua (6‐10 gestational weeks) and were characterized based on their phenotype and clonality. The immunophenotype and angiogenic capacity of dCD34^+^ cells were compared with BMSCs. And the therapeutic potential of the two kinds of cells was evaluated following their transplantation into IHD in rats.

## 2 MATERIALS AND METHODS

### 2.1 **Processing and culturing of human dCD34** ^+^ **cells and BMSCs**

Human decidual samples were collected from healthy women aged 20‐35 years old who underwent selective vaginal surgery to terminate early pregnancy. Informed consent was obtained from the study patients. All procedures performed in studies involving human participants were performed in accordance with the ethical standards of the Second Affiliated Hospital Research Ethics Committee of Harbin Medical University and conformed to the principles of the Declaration of Helsinki. Each sample was kept in phosphate buffered saline (PBS, pH 7.4) supplemented with 0.1% antibiotics (100 IU/mL penicillin and 100 mg/mL streptomycin; Beyotime, Shanghai, China) until it was processed in the laboratory (less than 2 hours later).

Each sample was washed three times in large volumes of sterile PBS containing 1% antibiotics (Beyotime). After that, the tissue was minced carefully into 1 to 2 mm^3^ pieces. Then, the tissue was incubated with 0.2% collagenase type IV (Biotopped, Beijing, China) and 0.25% trypsin‐ethylene diamine tetraacetic acid (trypsin‐EDTA; Gibco, Gaithersburg, MD, USA) for 60 minutes at 37°C with occasional shaking. The enzymatic reaction was stopped by adding Dulbecco's modified Eagle's medium and Nutrient Mixture F‐12 (DMEM/F12; HyClone, South Logan, Utah, USA) supplemented with 10% foetal bovine serum (FBS; Sciencell, San Diego, CA, USA). The cell suspensions were filtered through a nylon mesh (70 μm; BD Falcon, San Jose, CA, USA) to remove glands and clumps of epithelial cells. Referring to the manual for detailed procedures, the target cells were obtained with a CD34 MicroBead kit (Miltenyi Biotec, Auburn, CA, USA). The dCD34^+^ cells were resuspended in DMEM/F12 (HyClone) supplemented with 10% FBS (ScienCell) and then were seeded in a 25 cm^2^ culture flask (Nest, Wuxi, Jiangsu, China) at a concentration of 2.0 × 10^4^ cells/cm^2^; cells were maintained under 37°C, 5% CO_2_ and 95% humidity conditions. The culture medium was refreshed every third day.

Similarly, bone marrow was obtained from cardiac surgery patients (20‐35 years old) in the Second Affiliated Hospital of Harbin Medical University. BMSCs were isolated with a FicollPaque gradient (1.073 g/mL density; GE Healthcare, Little Chalfont, Buckinghamshire, UK) by centrifugation at 1330 × *g* for 20 minutes. The mononuclear cells were collected and rinsed twice, and then seeded in DMEM/F12 (HyClone) supplemented with 10% FBS (ScienCell) and cultured at 37°C in an atmosphere containing 5% CO_2_. Third‐generation BMSCs were harvested for the following experiments.

### 2.2 Immunohistochemistry

Immunohistochemistry staining was performed by a standard protocol. In brief, cryopreserved sections were fixed in 4% paraformaldehyde (PFA) for 10 minutes and blocked with 3% hydrogen peroxide (H_2_O_2_) for 10 minutes. Then, the sections were incubated first with rabbit anti‐human CD34 (1:100; Abcam, Cambridge, UK) for 12 hours and then with HRP‐conjugated goat anti‐rabbit (1:150; Wanleibio, Shenyang, Liaoning, China) for 30 minutes at room temperature. The samples were immersed in 3,3'‐diaminobenzidine for 10 minutes, after which they were stained with haematoxylin for 10 seconds. Finally, the samples were washed with PBS three times and then were photographed.

### 2.3 Flow cytometry

The decidual unsorted cells (dUCs; including dCD34^+^ and dCD34^‐^ cells) were collected and labelled with the following antibodies for dual staining: CD34‐FITC (BD, Franklin Lakes, NJ, USA)/ c‐kit‐PE (BD) for 30 minutes at 4°C. The dCD34^+^ cells were stained with CD34‐FITC (BD), c‐kit‐PE (BD), CD90‐FITC (BioLegend, San Diego, CA, USA), CD105‐APC (BioLegend), CD31‐FITC (BD), VEGFR‐2‐FITC (BD), VE‐cadherin‐FITC (BD), HLA‐ABC‐FITC (Abcam) and HLA‐DR‐FITC (Abcam). Then, the cells were washed three times with cold PBS before being centrifuged at 1000 rpm for 5 minutes. Immunoreactivity of the cell surface antibody markers was assayed by fluorescence‐activated cell sorting (FACS; BD).

### 2.4 Colony forming

The isolated dCD34^+^ cells were incubated at concentrations of 100, 1000 and 10 000 cells/cm^2^ in 6‐well plates (Nest) containing DMEM/F12 (HyClone) with 10% FBS (ScienCell), 25 μg/mL L‐ascorbic acid (Sigma, St Louis, MO, USA), 2 mmol/L L‐glutamine (Sigma), 200 μg/mL holotransferrin (Sigma), 50 ng/mL vascular endothelial growth factor (VEGF; Abcam), 10 ng/mL basic fibroblast growth factor (bFGF; Abcam) and 10 ng/mL interleukin‐6 (IL‐6; PeproTech, Rocky Hill, NJ, USA) for 15 days. Clusters of cells were considered colonies when they were visible to the naked eye and contained >20 cells.

### 2.5 Gene expression measurement

Total RNA was extracted directly from decidual unsorted cells (dUCs), dCD34^+^ cells and umbilical vein endothelial cells (UVECs; Control groups) using TRIzol reagent (Life Technologies, Carlsbad, CA, USA). Reverse transcription was performed using a PrimeScript™ RT reagent kit (Takara, Kusatsu, Shiga, Japan) according to the manufacturer's protocol. The gene expression levels of CD31, VE‐cadherin and VEGFR‐2 were determined by real‐time PCR (RT‐PCR) with 2× Taq Master Mix (Vazyme, Nanjing, Jiangsu, China) on a thermal cycler (S1000; Bio‐Rad, Hercules, CA, USA), which used a programme of 94°C for 2 minutes, followed by 35 cycles (94°C for 30 seconds, annealing temperature 60°C for 30 seconds and 72°C for 20 seconds). PCR products were separated on a 2% agarose gel by electrophoresis. The primers were designed and synthesized by Invitrogen (USA) as shown in Table 1.

**TABLE 1:** Primers used for real‐time PCR

Name	Sequence
CD31‐F	5′‐TGAGGTCAAAGGATCAGACGAC‐3′
CD31‐R	5′‐TGGTGGCAAGGGACTAAGGA‐3′
VE‐cadherin‐F	5′‐ATGAGATCGTGGTGGAAGCG‐3′
VE‐cadherin‐R	5′‐ATGTGTACTTGGTCTGGGTGA‐3′
VEGFR‐2‐F	5′‐TCTGCCTACCTCACCTGTTTC‐3′
VEGFR‐2‐R	5′‐TCTGCCTACCTCACCTGTTTC‐3′
GAPDH‐F	5′‐GTGGGATGCAACAGCCTTAGA‐3′
GAPDH‐R	5′‐CGCTCCTGGAAGATGGTGAT‐3′

### 2.6 Differentiation into endothelial‐like cells and paracrine effect

The dCD34^+^ cells and BMSCs were harvested and suspended in endothelial differentiation medium containing DMEM/F12 (HyClone), 10% FBS (ScienCell), 50 ng/mL VEGF (Abcam), 10 ng/mL bFGF (Abcam), 100 ng/mL endothelial cell growth supplement (ECGS; ScienCell), 1 ng/mL interleukin‐3 (IL‐3; PeproTech), 50 ng/mL interleukin‐11 (IL‐11; PeproTech) and 4.5 × 10^−4^ 1‐thioglycerol (Sigma). Cultures were incubated for 15 days. To detect endothelial function, cells (5.0 × 10^4^) were seeded in a 48‐well cell culture dish (Nest) coated with Matrigel matrix (BD). Endothelial differentiation was evaluated by indirect immunofluorescence staining for the expression of CD31 (Abcam) and vWF (Abcam).

For enzyme‐linked immunosorbent assay (ELISA), the supernatant of dCD34^+^ cells and BMSCs were collected after 24 hours of culture and analysed for VEGF and bFGF by ELISA kits (CUSABIO, Wuhan, Hubei, China) according to the manufacturer's protocol. Basal media were also measured as negative control.

### 2.7 Immunogenicity

To quantify leucocyte‐mediated cytotoxicity, peripheral blood leucocytes (5.0 × 10^5^) were isolated from healthy people and were cocultured with dCD34^+^ cells and BMSCs. Leucocyte‐mediated cytotoxicity was determined by evaluating lactate dehydrogenase (LDH) release from the damaged cells after 5 days of coculture. Lactate dehydrogenase (LDH) release reagent treatment was used as a positive control to test maximum LDH release. An LDH cytotoxicity assay kit (Beyotime) was used according to the manufacturer's protocol.

At the same time, leucocytes were stimulated with 5 μg/mL phytohemagglutinin (PHA; Sigma) and then cocultured with or without BMSCs and dCD34^+^ cells after 5 days of coculture. Proliferation of T cells was labelled with CD4‐APC (BD), CD8‐PE (BD) and measured by flow cytometry.

### 2.8 MI model and cell transplantation

All procedures performed in studies involving animals were in accordance with the ethical standards of the Guide for the Care and Use of Laboratory Animals and were approved by the Committee of Harbin Medical University. All adult male Sprague Dawley (SD) rats (200‐220 g) were acquired from the Animal Laboratory Center of the Second Affiliated Hospital of Harbin Medical University. Rats were given an intraperitoneal injection of cyclosporine A (5 mg/kg; Novartis, Basel, Switzerland) once a day from three days before surgery until the end of the experiment. In brief, rats were placed in the supine position for tracheal intubation (arterial puncture needle, 16G), and a ventilator (Harvard Apparatus, Medford, NJ, USA) was used for respiration. Through a left lateral thoracotomy, the anterior descending branch of the left coronary artery was ligated with a 6‐0 prolene needle at 2‐3 mm below the left atrial appendage. MI was confirmed by regional myocardial colour change. Fifteen minutes after MI, cells suspended in medium (2.0 × 10^6^/100 μL) were injected into one site at the centre and four sites to the boundary of the infarcted area. Then, the incision was closed. Rats were divided into three groups: culture medium, BMSC and dCD34^+^ cell transplantation group.

### 2.9 Cardiac function assessment

Cardiac function was measured by echocardiography in the three groups before and at 1 and 4 weeks after establishment of the MI model. After anaesthesia, animals were fixed in the left lateral position, and a 12‐MHz transducer (Vivid 7; GE Health care) was used to obtain an M‐ultrasound image. Then, the left ventricular end‐diastolic diameter (LVEDd), left ventricular end‐systolic diameter (LVESd), left ventricular ejection fraction (LVEF) and fractional shortening (LVFS) were measured. All echocardiography data were averaged from at least three consecutive cardiac cycles.

### 2.10 Infarct size measurement

Four weeks after transplantation, some animals were anesthetized, and median thoracotomy was performed as described above. Ten per cent potassium chloride (KCL) was perfused at the root of the aorta to ensure that the heart stopped at the diastolic phase. Then, the heart was quickly excised. A homemade balloon was placed into the left ventricle through the mitral valve and was fixed with suturing. The balloon was connected to a pressure detector, which was maintained at 20 mm Hg. The heart was fixed in 4% PFA for 1 week under these conditions and then was sectioned. The ratio of the sum of the length of the scar and the circumference was measured by Masson's trichrome, which defined the MI size of each of the myocardial surfaces. Final infarct size was expressed in average per cent from sections of each ventricle.

### 2.11 Infarct area angiogenesis

Four weeks after cell transplantation, the sections were used in immunohistochemistry experiments as follows. First, sections were incubated with rabbit anti‐rat a‐smooth muscle actin (a‐SMA, 1:400; Abcam) and CD31 (1:100; Abcam). Then, the density of arterioles and capillaries was counted by identifying positive cells per microscopic field (400× = 0.2 mm^2^) in five fields per sample and then averaging the results.

### 2.12 Survival of transplanted cells in vivo

One and four weeks after MI and cell transplantation, rats were sacrificed. The hearts were fixed in 4% PFA for 24 hours and then were dehydrated in 10%, 20% and 30% sucrose solutions for 1, 1 and 24 hours, respectively. The tissues were embedded in special moulds filled with optimal cutting temperature (OCT) compound (SAKURA, Torrance, CA, USA). The snap‐frozen tissues were cut into 5‐μm‐thick sections and then were incubated first with human‐specific anti‐mitochondria antibody (1:50; Millipore, Temecula, CA, USA) and then with goat anti‐mouse IgG‐Texas green (1:200; Santa Cruz, Dallas, TX, USA). The sections were immersed in 4′,6‐diamidino‐2‐phenylindole (DAPI; Sigma‐Aldrich) for 3 minutes to stain cell nuclei. The number of remaining cells was counted by identifying positive cells (blue nuclei surrounded by green cytoplasm) using immunofluorescence per microscopic field (400× = 0.2 mm^2^) in five fields per sample and then averaging the results.

### 2.13 Statistical analysis

Data represent the means ± standard deviation (SD) and were analysed with GraphPad Prism 5 software (GraphPad; La Jolla, CA, USA). Comparisons between two groups were performed using two‐tailed Student's t tests. One‐way ANOVA was used to determine the significance between three or more experimental groups. Statistical significance was considered at *P* < 0.05.

## 3 RESULTS

### 3.1 Presence of CD34^+^ precursors in human decidua

**FIGURE 1:**
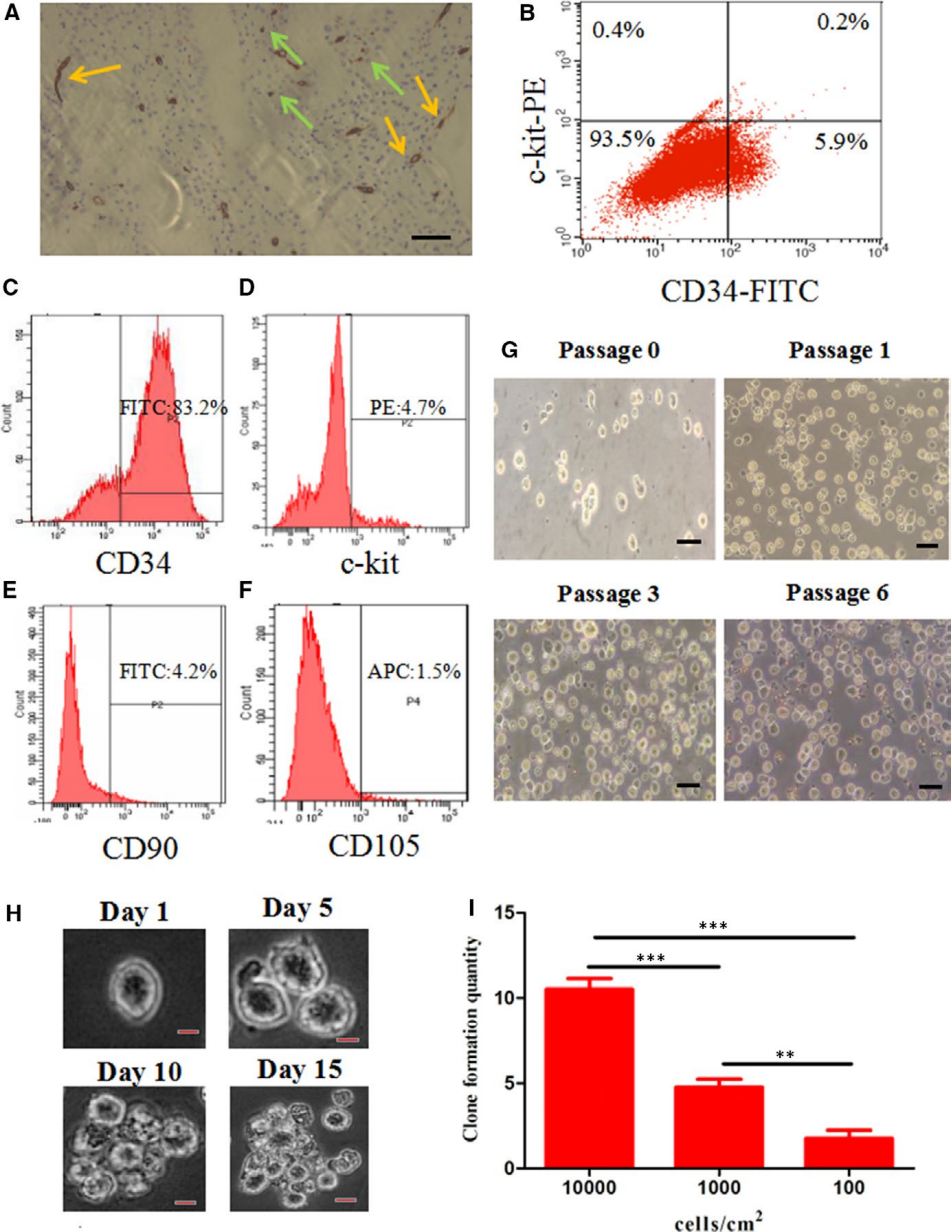
Discovery of CD34^+^ precursors in human decidua. A, Immunohistochemical analysis of decidual tissues and staining with CD34‐specific mAbs. The yellow arrows indicate CD34^+^ vessels. The green arrows indicate CD34^+^‐positive cells. B, Flow cytometry showed that the expression of CD34^+^/c‐kit^‐^ was found in 5.68 ± 0.66% of the cells in decidua. C, After bead sorting, 79.58 ± 3.77% of cells were CD34^+^. D, Most of the dCD34^+^ cells were c‐kit^‐^. E,F, dCD34^+^ cells hardly expressed mesenchymal cell‐specific markers CD90 and CD105. G, Individual dCD34^+^ cells had round shapes, and the boundaries were clear. H, Morphology of a representative colony derived from dCD34^+^ cells over 15 d. I, Clonal quantities increased with the cell inoculation densities (ANOVA; **P < 0.01; ***P < 0.001; and n = 4/group). Scale bars in (A,G) represent 100 μm; in H, it represents 50 μm

Immunohistochemistry was used to assess the location of dCD34^+^ cells. A CD34‐specific antibody provided strong staining of vascular structures (Figure 1A). However, other single dCD34^+^ cells were scattered in the decidua, and these were the target cells (Figure 1A, n = 4/group). The results showed that the dCD34^+^ cells were distributed from the surface and the bottom layer of the tissues. In view of this finding, unsorted cell suspensions isolated from human decidua were analysed for the surface expression of CD34 and c‐kit antigen. Flow cytometry showed that CD34^+^/c‐kit^‐^ cells were present in the decidua at the first trimester, and this expression pattern was found in 5.68 ± 0.66% of cells (Figure 1B, n = 4/group).

### 3.2 Isolation of dCD34^+^ cells

Flow cytometry showed that the purity of dCD34^+^ cells after magnetic bead sorting was 79.58 ± 3.77% (Figure 1C, n = 4/group), and the majority of them hardly expressed c‐kit, CD90 and CD105 (Figure 1D‐F). Under an inverted microscope, the cells appeared round shape with centrally located nuclei and were in a suspended state (Figure 1G). After a series of passages, they retained their typical morphology and proliferation activity.

### 3.3 Clonal formation of dCD34^+^ cells

Clonogenicity of the cells is considered to be a major characteristic of their growth potential. After 15 days, colonies could be formed (Figure 1H). dCD34^+^ cells at third passage were collected and seeded at concentrations of 100, 1000 and 10 000 cells/cm^2^. The average cloning formation values for the three concentrations were determined to be 10.5 ± 1.3 for 10 000 cells/cm^2^, 4.8 ± 1.0 for 1000 cells/cm^2^ and 1.8 ± 1.0 for 100 cells/cm^2^, respectively (Figure 1I, n = 4/group).

### 3.4 No endothelial genes are expressed in dCD34^+^ cells

**FIGURE 2:**
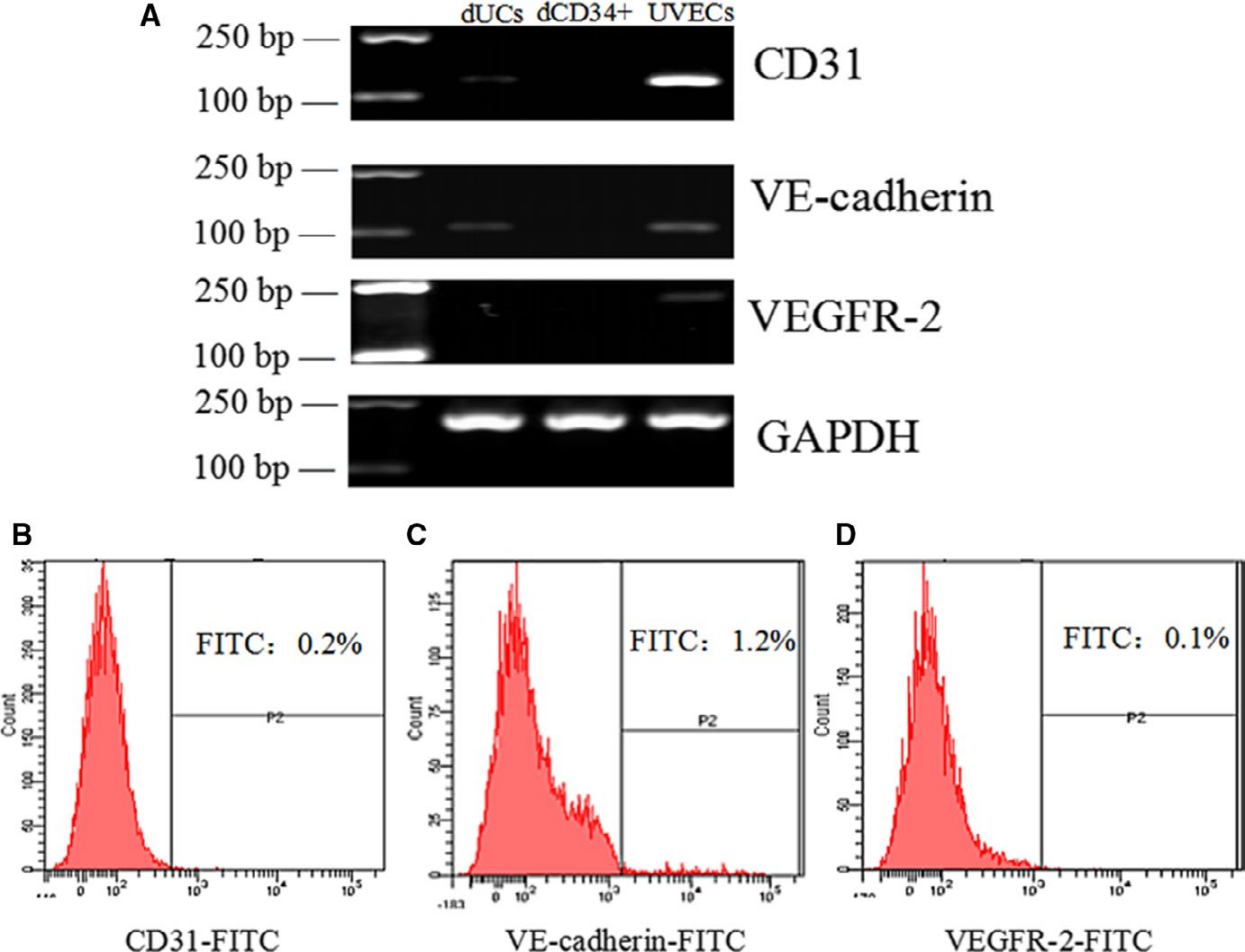
Validation of non‐vascular endothelial cells. A, CD31, VE‐cadherin and VEGFR‐2 were strongly expressed in UVECs, CD31 and VE‐cadherin were weakly expressed in dUCs, and all were unexpressed in dCD34^+^ cells. B‐D, dCD34^+^ cells did not express the above three characteristic endothelial cell markers, as assessed by FACS (n = 4/group)

The endothelial cell‐specific genes CD31, VE‐cadherin and VEGFR‐2 were detected by RT‐PCR. The results showed that the dCD34^+^ cells did not express CD31, VE‐cadherin or VEGFR‐2. The unsorted decidual cells weakly expressed CD31 and VE‐cadherin. Meanwhile, UVECs expressed all of these genes more strongly than the dCD34^+^ cells did (Figure 2A, n = 4/group). Apart from this, flow cytometry showed that the dCD34^+^ cells also did not express CD31, VE‐cadherin or VEGFR‐2 (Figure 2B‐D).

### 3.5 Angiogenesis and paracrine effect of dCD34^+^ cells

**FIGURE 3:**
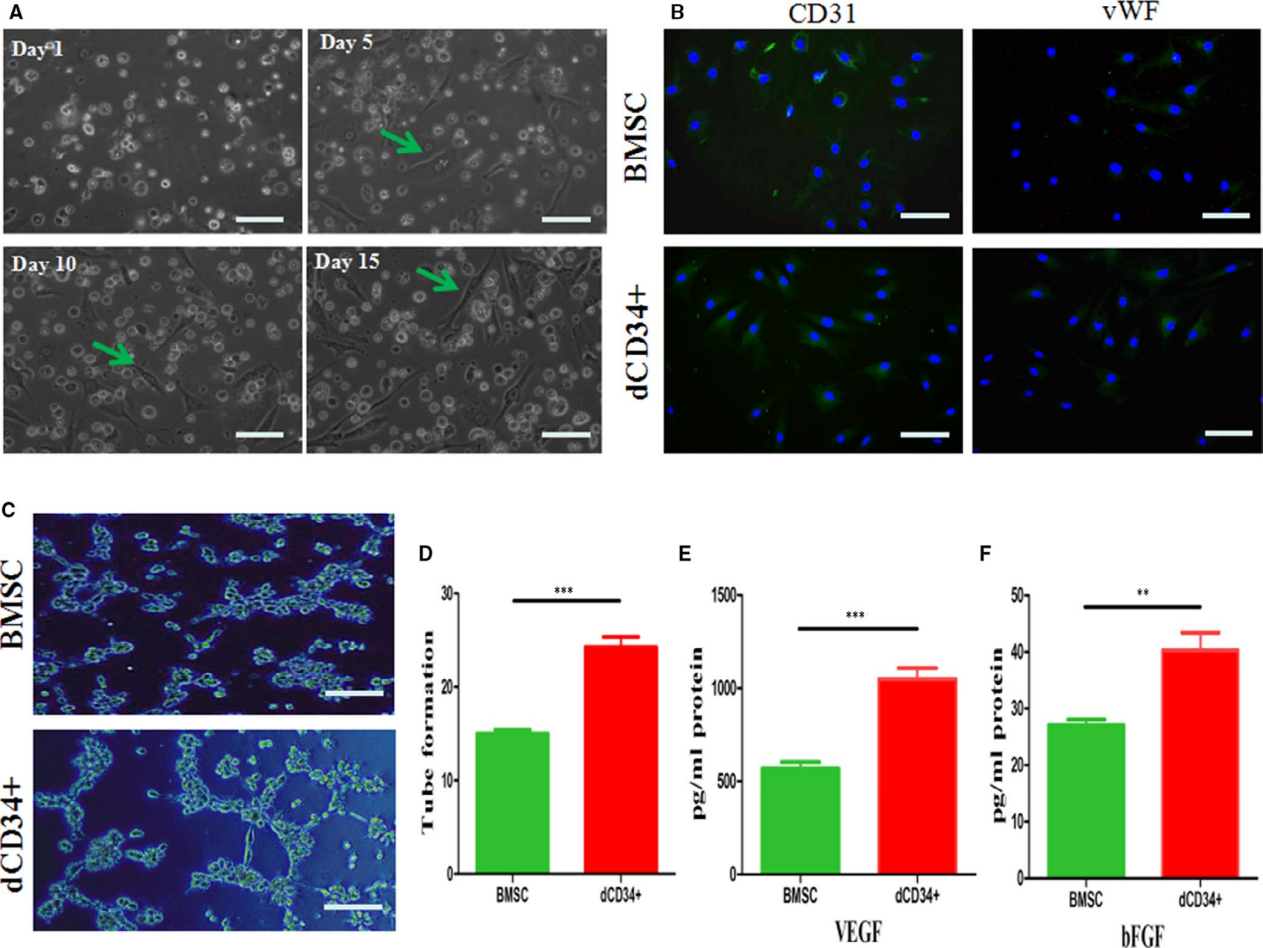
Analysis of angiogenesis and paracrine effect in dCD34^+^ cells. A, dCD34^+^ cells began to differentiate into adherent cells (green arrow) in 5 d, and increasing numbers of adherent cells appeared until 15 d. B, Induced dCD34^+^ cells (adherent) and BMSCs could express CD31 and vWF, as shown by immunofluorescence staining. (C,D) Tube formation of dCD34^+^ cells (adherent) and BMSCs was assessed in Matrigel. dCD34^+^ cells formed more tubule structures than BMSCs did. (E,F) In vitro release of VEGF, bFGF from dCD34^+^ cells compared with BMSCs (two‐tailed t test; **P < 0.05; ***P < 0.001; and n = 4/group). Scale bars in (A‐C) represent 100 μm

After 5 days of induced differentiation, both dCD34^+^ cells and BMSCs could be differentiated into adherent cells (Figure 3A). After staining with an antibody against CD31 and vWF, positive bright green fluores**c**ence was observed within the adherent cells and BMSCs (Figure 3B). In Matrigel tube formation experiments, the induced adherent cells were shown to form tube structures (Figure 3C). More tube structures were formed by the dCD34^+^ cells (adherent) than were formed by the BMSCs over the same amount of time (Figure 3D, n = 4/group).

In addition, to characterize dCD34^+^ cells and BMSCs release of cytokines (VEGF and bFGF), the supernatant of cells was collected after 24 hours of culture. With the measurement of ELISA, the release of VEGF (1049 pg/mL protein from dCD34^+^ cells versus 568 pg/mL protein from BMSCs) and bFGF (40 pg/mL vs 27 pg/mL) (Figure 3E,F, n = 4/group). Cytokines were not detected in basal culture media.

### 3.6 Immunogenicity of dCD34^+^ cells

**FIGURE 4:**
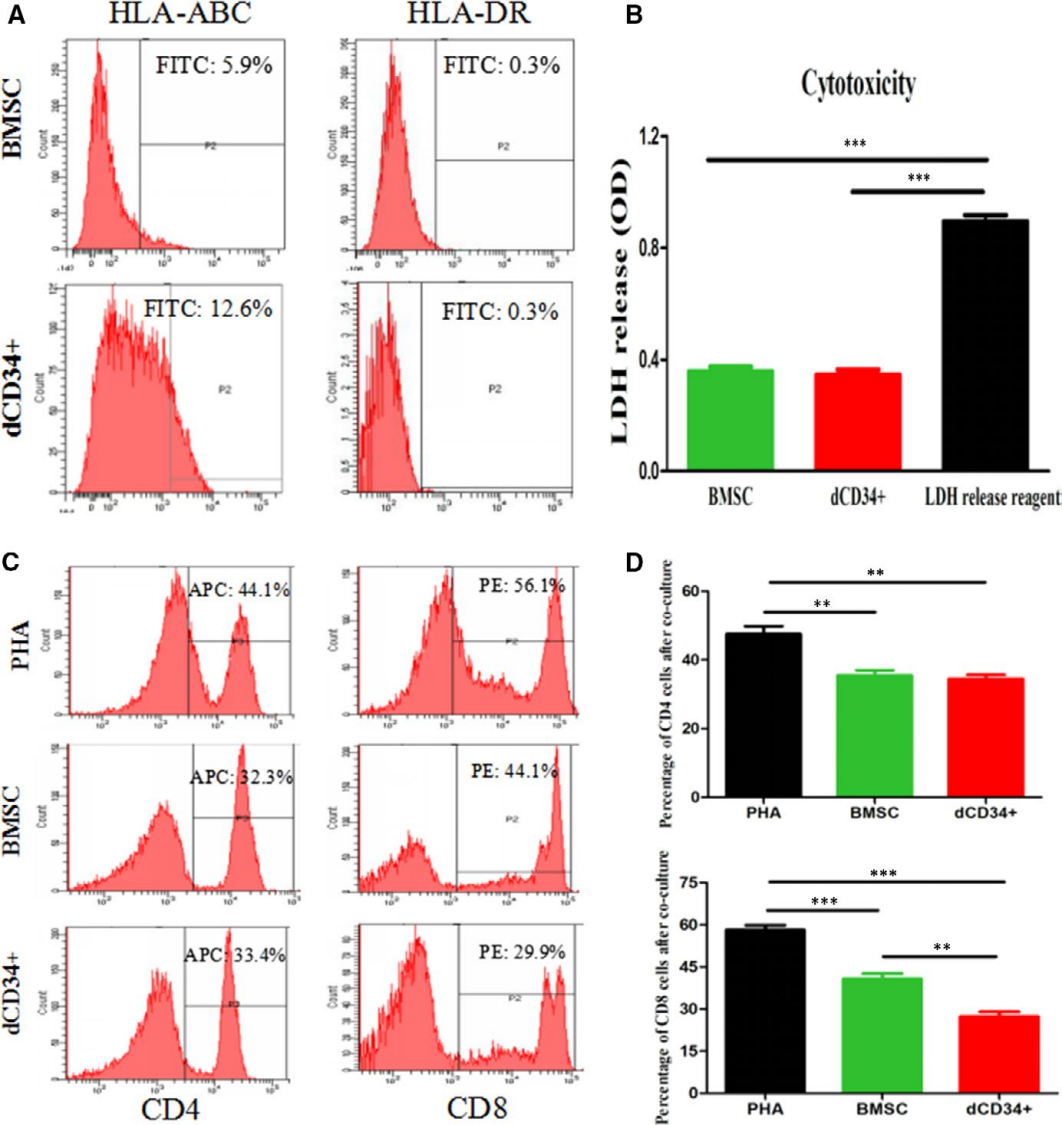
Immunological characteristics of dCD34^+^ cells. A, Flow cytometry showed that dCD34^+^ cells weakly expressed HLA‐ABC and hardly expressed HLA‐DR; the results were the same in BMSCs. B, Almost the same cytotoxicity was observed in dCD34^+^ cells and BMSCs. LDH release reagent treatment was used as a positive control of LDH release, which was much higher than the other two groups (ANOVA; ***P < 0.001; and n = 4/group). (C,D) After 5 d of coculture, the lymphocytes of three groups were removed and stained for CD4 and CD8, then measured by flow cytometry (ANOVA; **P < 0.05; ***P < 0.001; and n = 4/group)

To measure immunological surface markers, flow cytometry was performed. The results showed that dCD34^+^ cells had lower expression of HLA‐ABC and hardly expressed HLA‐DR, which was similar to what was observed in BMSCs (Figure 4A, n = 4/group). To evaluate immune properties, we established a coculture system with allogeneic leucocytes, generating a mixed leucocyte reaction (MLR) in vitro. After 5 days of coculture, the level of cytotoxicity assessed by LDH release was almost the same in dCD34^+^ cells and in BMSCs (Figure 4B, n = 4/group).

Flow cytometry was used to further confirm the effect of dCD34^+^ cells and BMSCs on the proliferation of PHA‐activated T cells. Our data revealed that dCD34^+^ cells and BMSCs could suppress the proliferation of CD4 and CD8 T cells compared with the positive controls (only PHA‐induced). And the proliferation of CD4 T cells in dCD34^+^ cells group was almost the same level as BMSCs group and CD8 T cells were higher in BMSCs group compared with dCD34^+^ cells group (Figure 4C,D, n = 4/group).

### 3.7 Infarct size decreases after transplantation of dCD34^+^ cells

**FIGURE 5:**
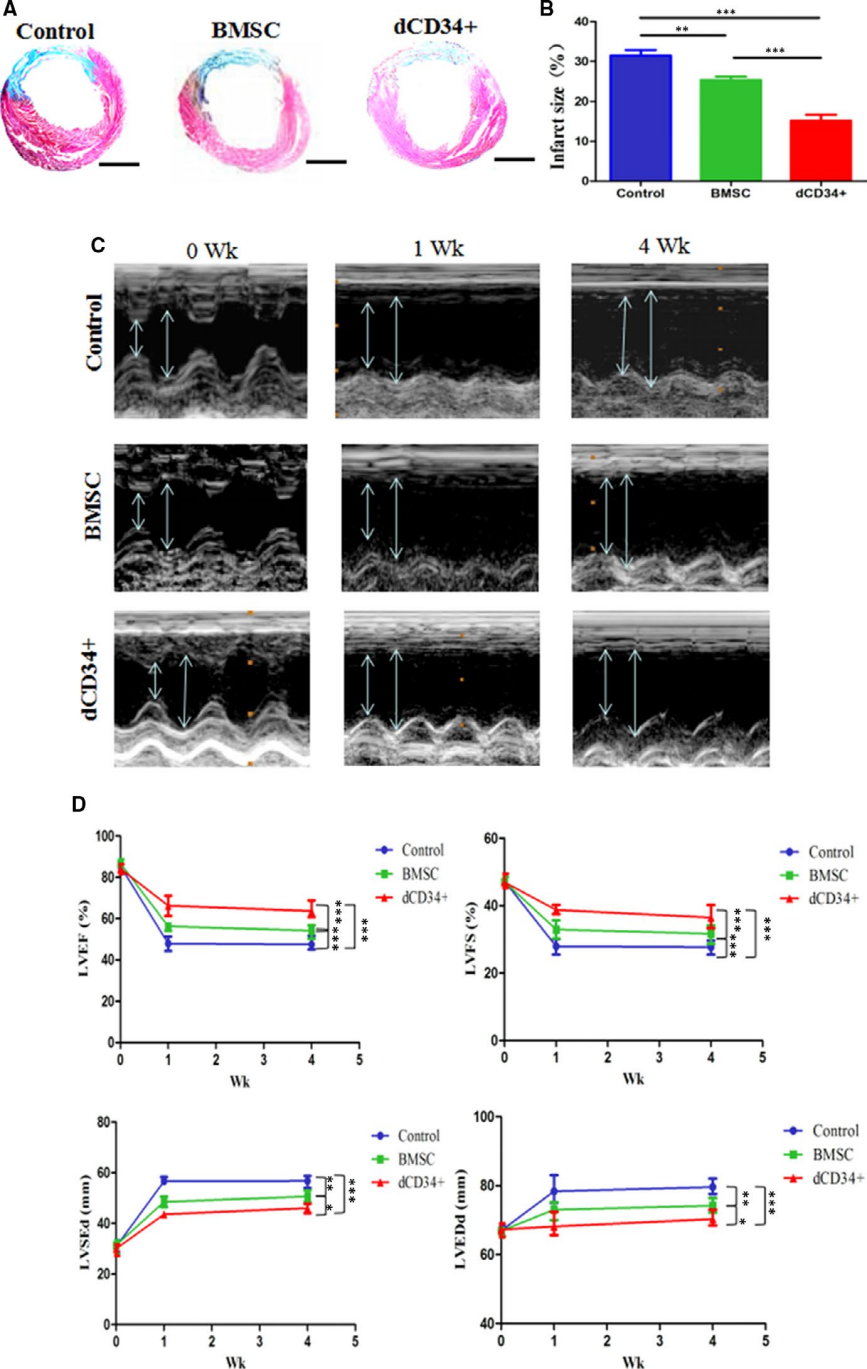
Cell therapy for IHD. A, Masson's trichrome staining of the infarct size 4 wk after cell transplantation in the three groups (blue = collagen; red = myocardium). Scale bars represent 2 mm. B, The infarct size of the dCD34^+^ group was significantly smaller than that of the medium control and BMSC group 4 wk after MI, and the infarct size of the BMSC group was also significantly smaller than that of the control. C, Representative echocardiography images from the three groups before and 1 and 4 wk after MI (high lines: LVESd; low lines: LVEDd). D, The LVEF and LVFS of the dCD34^+^ group after MI were significantly higher than those of the control and BMSC groups. The LVESd and LVEDd of the dCD34^+^ group after MI were significantly smaller than those of the control and BMSC groups. The same results were observed in the BMSC group compared to the control (ANOVA; *P < 0.05, **P < 0.01, ***P < 0.001; and n = 6/group)

Four weeks after MI and cell therapy, computerized morphometric analysis demonstrated that the infarct size was significantly smaller in dCD34^+^ cells and BMSCs group than it was in control group (Figure 5A,B, n = 6/group). In accordance with the data, the infarct size was also markedly reduced in dCD34^+^ cells group compared with BMSCs group. This result indicated that the dCD34^+^ group could effectively reverse ventricular remodelling after MI.

### 3.8 Cardiac function is improved following transplantation of dCD34^+^ cells

One and four weeks after MI, echocardiography was performed. The LVEF and LVFS of the dCD34^+^ and BMSCs transplantation group were significantly higher than the control group, while the LVEDd and LVSDd were significantly lower than the control group (Figure 5C,D, n = 6/group). And the above information was identical in dCD34^+^ group compared with BMSCs group. This indicated that dCD34^+^ cells could improve cardiac function after MI.

### 3.9 More survival and neovascularization occurs following transplantation of dCD34^+^ cells

**FIGURE 6:**
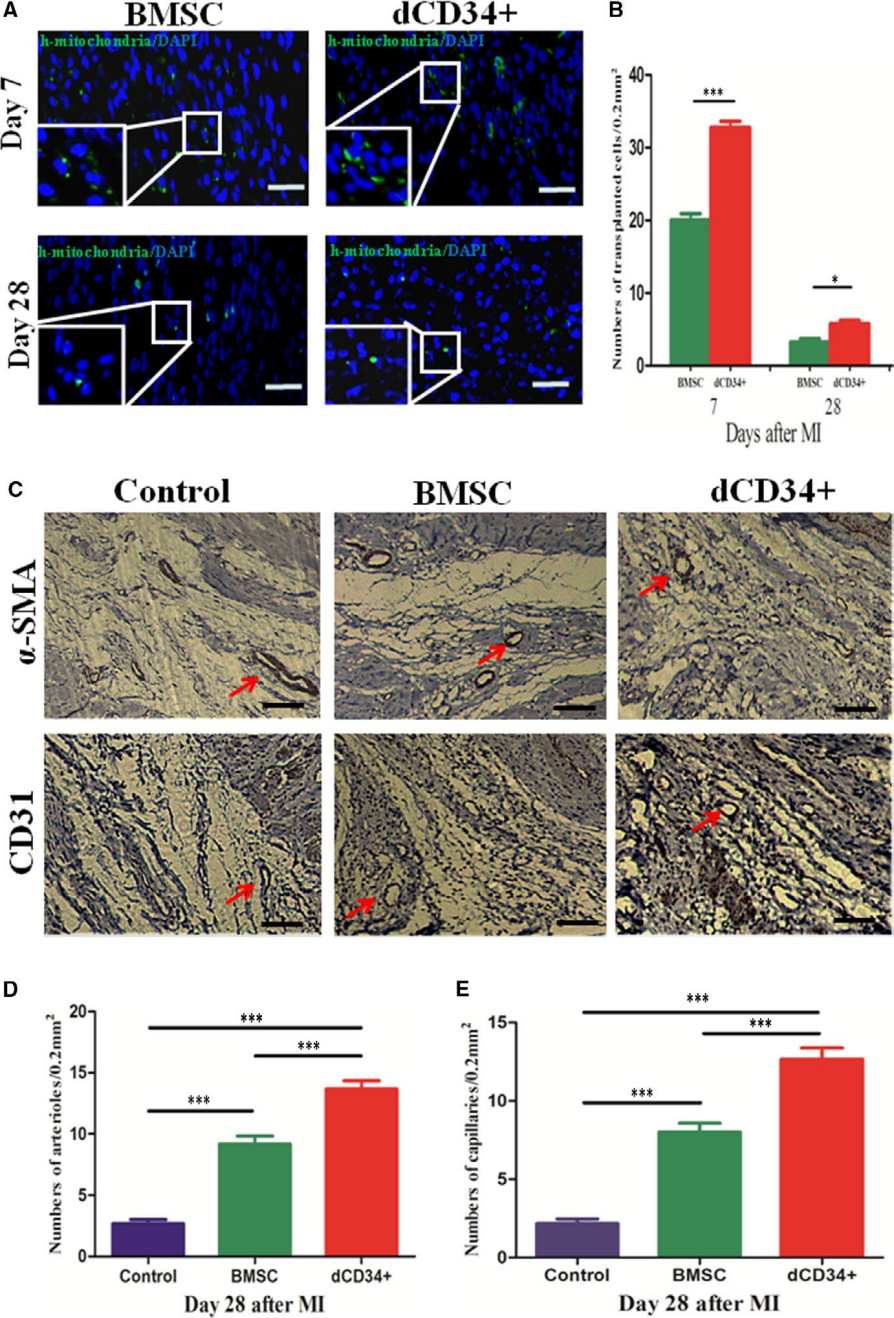
Cell survival and neovascularization. A, Anti‐human mitochondrial staining showed the survival of transplanted cells. B, The number of dCD34^+^ cells that survived was significantly greater than that in the BMSC group 7 and 28 d after cell transplantation. C, Staining for a‐SMA and CD31 (red arrows) was used to visualize the blood vessel density. (D,E) Arteriole and capillary densities were higher in the dCD34^+^ group than they were in the other two groups at 28 d after MI. The same results were observed in the BMSC group and in the control (ANOVA; *P < 0.05, ***P < 0.001; and n = 6/group). Scale bars in (A,C) represent 100 μm

Immunofluorescence of mitochondrial stain and immunohistochemistry were used to detect cell survival and neovascularization in the infarcted area, respectively. As a result, the cells in the dCD34^+^ group survived at 1 and 4 weeks, and the formation of arterioles and capillary density at 4 weeks were higher than those of the other two groups (Figure 6A‐D, n = 6/group). It showed that dCD34^+^ cells had a strong antiapoptotic and angiogenic capacity, which could significantly improve blood supply in the MI area.

## 4 DISCUSSION

Menstrual shedding and subsequent repair of the surface endometrium are involved in massive changes in vessels, which are related to the stem cells of the endometrium.^17^, ^18^, ^19^ Stimulated by progesterone, the endometrial stroma differentiates into the decidua in the first trimester.^20^, ^21^ This rapidly changing process also depends on the formation of numerous vessels, which provide sufficient nutrients for the embryos at the same time.^9^ Therefore, angiogenic stem cells may be present in the decidua. Previous studies have shown that mouse uterine‐derived CD34^+^ cell populations are hemangioblasts.^16^ CD34 is a marker of vascular endothelial progenitor cells, which indicates that CD34^+^ cell populations are involved in vessel formation.^13^ Therefore, we speculated that human dCD34^+^ cells in the first trimester may be angiogenic stem cells.

By immunohistochemical staining of the decidua, we found that CD34 was expressed not only in decidual vascular endothelial cells but also in single cells (dCD34^+^ cells). Most dCD34^+^ cells were found in the surface and bottom layers of the decidua. Therefore, we found the target cell in the decidua. After 24 hours of culture, one‐fifth of dUCs became adherent. Flow cytometry showed that the majority of adherent cells was CD34^‐^ MSCs (data not shown). Next, we sorted the suspension cells with CD34^+^ magnetic beads. We found that the purity of the dCD34^+^ cells was 79.58 ± 3.77%, and the majority of them were c‐kit^‐^. This result was consistent with that of the hemangioblast population (CD34^+^/c‐kit^‐^) in the mouse uterus.^16^ Hence, we found that dCD34^+^ cells may be capable of an angiogenic phenotype. In addition, we found that dCD34^+^ cells were not consistent with the growth morphology of mesenchymal cells and endothelial cells. In addition, the MSC surface markers CD90 and CD105,^3^, ^22^ and endothelial cell‐specific genes CD31, VE‐cadherin and VEGFR‐2 were hardly expressed.^23^, ^24^ Further conformed that dCD34^+^ cells were not MSCs and endothelial cells.

Stem cells have been characterized by their ability to be passaged while maintaining clonality and differentiation potential.^10^, ^15^, ^16^, ^25^, ^26^ Our research found that dCD34^+^ cells could be stably passaged for at least 6 passages. The cells were cultured in clone medium for 5 days and then began to form clusters. By the 15th day, colonies could be formed. Furthermore, dCD34^+^ cells were added to a suitable concentration of endothelial cell differentiation solution for 5 days, and some of the suspended cells adhered. We found that the cell morphology became similar to that of endothelial cells, and they could form more tube structures in the Matrigel than BMSCs during the same amount of time. Above all, we confirmed for the first time that dCD34^+^ cells were angiogenic stem cells, which had angiogenic properties that were significantly improved over those of BMSCs.

HLA‐ABC and HLA‐DR belong to the classical HLA‐I and HLA‐II complexes, which are key molecules involved in antigen processing, treatment and presentation.^27^ Studies have shown that immunoprivileged cells hardly express HLA‐I and HLA‐II, which allow them to be tolerated in allogeneic transplantation.^28^, ^29^ Our results showed that dCD34^+^ cells weakly expressed HLA‐ABC and hardly expressed HLA‐DR. In addition, coculture of MSCs with allogeneic lymphocytes failed to stimulate lymphocyte proliferation, indicating that MSCs were not innately immunogeneic.^30^, ^31^ We found that dCD34^+^ cells exhibited cytotoxicity towards leucocytes that were almost the same as that of BMSCs. CD4 and CD8 T cells are among the most important population of immune cells, which have been shown to be the major cause of allogeneic graft rejection.^32^ Reports suggest that MSCs can suppress CD4 and CD8 T cells proliferation.^33^ We confirmed that coculture of dCD34^+^ cells could significantly suppress CD4^+^ T cells proliferation in a similar degree with BMSCs and better than BMSCs in the suppression of CD8^+^ T cells proliferation. The above discussion suggests that dCD34^+^ cells have low immunogenicity.

Before menopause, the number of women suffering from IHD is lower than the number of men. Studies have shown that uterus‐derived cells home to the damaged heart, which could improve cardiac function.^34^ This showed that uterine‐derived cells could be useful for IHD. Our study found that the dCD34^+^ cell group had a more significant effect on improving cardiac function, reducing the MI area and forming more vessels to increase the blood supply in the infarct area than the BMSC group. This may be related to the stronger angiogenic and antiapoptotic ability of dCD34^+^ cells. In addition, some studies have suggested that the paracrine function also plays a vital role after transplantation.^35^ Our data demonstrated that dCD34^+^ cells could secrete angiogenic cytokines (VEGF and bFGF) in vitro and stimulate neovascularization in vivo. It showed the importance of paracrine effects in mediating tissue responses to ischaemia.

## 5 CONCLUSION

We report for the first time that the human decidua from the first trimester contains CD34^+^ angiogenic stem cells, which have strong angiogenic properties and low immunogenicity in vitro. After cell transplantation in MI rats, the dCD34^+^ cell group was shown to have therapeutic effects for IHD that were superior to those of BMSCs. Therefore, dCD34^+^ cells are likely to be an excellent seed cell for IHD in the field of regenerative medicine.

## CONFLICT OF INTEREST

All the authors declare no conflict of interest.

## AUTHOR CONTRIBUTION

**Long Bai:** Data curation (lead); Formal analysis (lead); Methodology (lead); Resources (lead); Software (lead); Validation (lead); Writing‐original draft (lead). **Lu Sun:** Data curation (lead); Formal analysis (lead); Funding acquisition (supporting); Project administration (lead); Supervision (lead); Visualization (lead); Writing‐review & editing (lead). **Wei Chen:** Conceptualization (supporting); Data curation (lead); Investigation (lead); Supervision (lead). **Kai‐Yu Liu:** Conceptualization (supporting); Data curation (lead); Methodology (equal); Resources (lead); Supervision (supporting). **Chun‐Feng Zhang:** Data curation (lead); Investigation (equal); Supervision (equal). **Fei Wang:** Resources (supporting); Software (lead). **Gui‐Huan Zhang:** Data curation (equal); Formal analysis (equal); Investigation (equal); Methodology (equal); Resources (equal). **Ye Huang:** Data curation (equal); Formal analysis (equal); Investigation (equal); Resources (equal). **Jing‐Xuan Li:** Data curation (equal); Formal analysis (equal); Investigation (equal); Resources (equal). **Ying Gao:** Data curation (equal); Formal analysis (equal); Investigation (equal). **Xin Sun:** Data curation (equal); Investigation (equal). **Wei Liu:** Data curation (supporting); Methodology (supporting). **Guo‐Qing Du:** Software (supporting). **Ren‐Ke Li:** Conceptualization (supporting); Supervision (supporting); Writing‐review & editing (supporting). **Ming‐Li Huang:** Conceptualization (lead); Data curation (supporting); Funding acquisition (supporting); Methodology (supporting); Project administration (supporting); Resources (lead); Supervision (lead); Writing‐review & editing (supporting). **Hai Tian:** Conceptualization (lead); Funding acquisition (lead); Investigation (lead); Methodology (lead); Project administration (lead); Resources (lead); Supervision (lead); Validation (lead); Writing‐review & editing (lead).

## Supporting information

Fig S1

## Data Availability

All data used to support the findings of this study are available from the corresponding author upon request.

## References

[1] Wang X‐Q , Shao Y , Ma C‐Y , et al. Decreased SIRT3 in aged human mesenchymal stromal/stem cells increases cellular susceptibility to oxidative stress. J Cell Mol Med. 2014;18:2298‐2310. 25210848 10.1111/jcmm.12395 PMC4224562

[2] Yao J , Jiang S‐L , Liu W , et al. Tissue inhibitor of matrix metalloproteinase‐3 or vascular endothelial growth factor transfection of aged human mesenchymal stem cells enhances cell therapy after myocardial infarction. Rejuvenation Res. 2012;15:495‐506. 22950427 10.1089/rej.2012.1325 PMC3482878

[3] Wu H , Li J‐Z , Xie B‐D , et al. Lower senescence of adipose‐derived stem cells than donor‐matched bone marrow stem cells for surgical ventricular restoration. Stem Cells Dev. 2018;27:612‐623. 29630447 10.1089/scd.2017.0271

[4] Kim YJ , Kim HK , Cho HH , et al. Direct comparison of human mesenchymal stem cells derived from adipose tissues and bone marrow in mediating neovascularization in response to vascular ischemia. Cell Physiol Biochem. 2007;20:867‐876. 17982269 10.1159/000110447

[5] Lu D , Liao Y , Zhu S‐H , et al. Bone‐derived Nestin‐positive mesenchymal stem cells improve cardiac function via recruiting cardiac endothelial cells after myocardial infarction. Stem Cell Res Ther. 2019;10:1–15. 31029167 10.1186/s13287-019-1217-x PMC6487029

[6] Trounson A , McDonald C . Stem cell therapies in clinical trials: progress and challenges. Cell Stem Cell. 2015;17:11‐22. 26140604 10.1016/j.stem.2015.06.007

[7] de Almeida FM , de Matos Branco AD , Fernandes‐Platzgummer A , et al. Addressing the manufacturing challenges of cell‐based therapies. Adv Biochem Eng Biotechnol. 2020;171:225‐278. 31844924 10.1007/10_2019_118

[8] Gao F , Bian F , Ma X , et al. Control of regional decidualization in implantation: Role of FoxM1 downstream of Hoxa10 and cyclin D3. Sci Rep. 2015;5:1–16. 10.1038/srep13863 PMC4563553 26350477

[9] Hess AP , Hamilton AE , Talbi S , et al. Decidual stromal cell response to paracrine signals from the trophoblast: amplification of immune and angiogenic modulators. Biol Reprod. 2007;76:102‐117. 17021345 10.1095/biolreprod.106.054791

[10] Dimitrov R , Kyurkchiev D , Timeva T , et al. First‐trimester human decidua contains a population of mesenchymal stem cells. Fertil Steril. 2010;93:210‐219. 19006798 10.1016/j.fertnstert.2008.09.061

[11] Nielsen JS , McNagny KM . Novel functions of the CD34 family. J Cell Sci. 2008;121:3683‐3692. 18987355 10.1242/jcs.037507

[12] Sidney LE , Branch MJ , Dunphy SE , et al. Concise review: evidence for CD34 as a common marker for diverse progenitors. Stem Cells. 2014;32:1380‐1389. 24497003 10.1002/stem.1661 PMC4260088

[13] Tasev D , Konijnenberg LSF , Amado‐Azevedo J , et al. CD34 expression modulates tube‐forming capacity and barrier properties of peripheral blood‐derived endothelial colony‐forming cells (ECFCs). Angiogenesis. 2016;19:325‐338. 27043316 10.1007/s10456-016-9506-9 PMC4930476

[14] Li T , Ma Q , Ning M , et al. Cotransplantation of human umbilical cord‐derived mesenchymal stem cells and umbilical cord blood‐derived CD34^+^ cells in a rabbit model of myocardial infarction. Mol Cell Biochem. 2014;387:91‐100. 24166198 10.1007/s11010-013-1874-5

[15] Yeh ET , Zhang S , Wu HD , et al. Transdifferentiation of human peripheral blood CD34^+^‐enriched cell population into cardiomyocytes, endothelial cells, and smooth muscle cells in vivo. Circulation. 2003;108:2070‐2073. 14568894 10.1161/01.CIR.0000099501.52718.70

[16] Sun Z , Zhang Y , Brunt KR , et al. An adult uterine hemangioblast: evidence for extramedullary self‐renewal and clonal bilineage potential. Blood. 2010;116:2932‐2941. 20606165 10.1182/blood-2010-01-266882

[17] Garry R , Hart R , Karthigasu KA , et al. A re‐appraisal of the morphological changes within the endometrium during menstruation: a hysteroscopic, histological and scanning electron microscopic study. Hum Reprod. 2009;24:1393‐1401. 19252193 10.1093/humrep/dep036

[18] Gargett CE , Chan RW , Schwab KE . Hormone and growth factor signaling in endometrial renewal: role of stem/progenitor cells. Mol Cell Endocrinol. 2008;288:22‐29. 18403104 10.1016/j.mce.2008.02.026

[19] De Carvalho Rodrigues D , Asensi KD , Vairo L , et al. Human menstrual blood‐derived mesenchymal cells as a cell source of rapid and efficient nuclear reprogramming. Cell transplant. 2012;21:2215‐2224. 22776164 10.3727/096368912X653048

[20] Ramathal C , Bagchi I , Taylor R , et al. Endometrial decidualization: of mice and men. Semin Reprod Med. 2010;28:17‐26. 20104425 10.1055/s-0029-1242989 PMC3095443

[21] Maruyama T , Yoshimura Y . Molecular and cellular mechanisms for differentiation and regeneration of the uterine endometrium. Endocr J. 2008;55:795‐810. 18580040 10.1507/endocrj.k08e-067

[22] Araújo AB , Salton GD , Furlan JM , et al. Comparison of human mesenchymal stromal cells from four neonatal tissues: amniotic membrane, chorionic membrane, placental decidua and umbilical cord. Cytotherapy. 2017;19:577‐585. 28343898 10.1016/j.jcyt.2017.03.001

[23] Zhou L , Lu M , Zhong W , et al. Low‐dose docetaxel enhances the anti‐tumour efficacy of a human umbilical vein endothelial cell vaccine. Eur J Pharm Sci. 2020;142:105163. 31756447 10.1016/j.ejps.2019.105163

[24] Pulous FE , Grimsley‐Myers CM , Kansal S , et al. Talin‐dependent integrin activation regulates VE‐cadherin localization and endothelial cell barrier function. Circ Res. 2019;124:891‐903. 30707047 10.1161/CIRCRESAHA.118.314560 PMC6521868

[25] Behfar A , Yamada S , Crespo‐Diaz R , et al. Guided cardiopoiesis enhances therapeutic benefit of bone marrow human mesenchymal stem cells in chronic myocardial infarction. J Am Coll Cardiol. 2010;56:721‐734. 20723802 10.1016/j.jacc.2010.03.066 PMC2932958

[26] Du X , Yuan Q , Qu YE , et al. Endometrial mesenchymal stem cells isolated from menstrual blood by adherence. Stem Cells Int. 2016;2016:3573846. 26681948 10.1155/2016/3573846 PMC4670906

[27] Ibrahim EC , Guerra N , Lacombe MJ , et al. Tumor‐specific up‐regulation of the nonclassical class I HLA‐G antigen expression in renal carcinoma. Cancer Res. 2001;61:6838‐6845. 11559559

[28] Tsuji H , Ikegami Y , Miyoshi S , et al. Xenografted human amniotic membrane‐derived mesenchymal stem cells acquired immune tolerance and transdifferentiated into cardiomyocytes in vivo. Circ Res. 2010;106:1613‐1623. 20508201 10.1161/CIRCRESAHA.109.205260

[29] Chang C‐J , Yen M‐L , Chen Y‐C , et al. Placenta‐derived multipotent cells exhibit immunosuppressive properties that are enhanced in the presence of interferon‐γ. Stem Cells. 2006;24:2466‐2477. 17071860 10.1634/stemcells.2006-0071

[30] Le Blanc K , Rasmusson I , Sundberg B , et al. Treatment of severe acute graft‐versus‐host disease with third party haploidentical mesenchymal stem cells. Lancet. 2004;363:1439‐1441. 15121408 10.1016/S0140-6736(04)16104-7

[31] Kode JA , Mukherjee S , Joglekar MV , et al. Mesenchymal stem cells: immunobiology and role in immunomodulation and tissue regeneration. Cytotherapy. 2009;11:377‐391. 19568970 10.1080/14653240903080367

[32] Ludke A , Wu J , Nazari M , et al. Uterine‐derived progenitor cells are immunoprivileged and effectively improve cardiac regeneration when used for cell therapy. J Mol Cell Cardiol. 2015;84:116‐128. 25939780 10.1016/j.yjmcc.2015.04.019

[33] Di Nicola M , Carlo‐Stella C , Magni M , et al. Human bone marrow stromal cells suppress T‐lymphocyte proliferation induced by cellular or nonspecific mitogenic stimuli. Blood. 2002;99:3838‐3843. 11986244 10.1182/blood.v99.10.3838

[34] Xaymardan M , Sun Z , Hatta K , et al. Uterine cells are recruited to the infarcted heart and improve cardiac outcomes in female rats. J Mol Cell Cardiol. 2012;52:1265‐1273. 22446160 10.1016/j.yjmcc.2012.03.002

[35] Sanz‐Ruiz R , Gutiérrez Ibañes E , Arranz AV , et al. Phases I‐III clinical trials using adult stem cells. Stem Cells Int. 2010;2010:579142. 21076533 10.4061/2010/579142 PMC2975079

