# Supplementary figures and images for: Evidence for the existence of CD34+ angiogenic stem cells in human first‐trimester decidua and their therapeutic for ischaemic heart disease
Source: J Cell Mol Med. 2020 Sep 8;24(20):11837–48. doi: 10.1111/jcmm.15800 (PMC7578869; doi:10.1111/jcmm.15800)

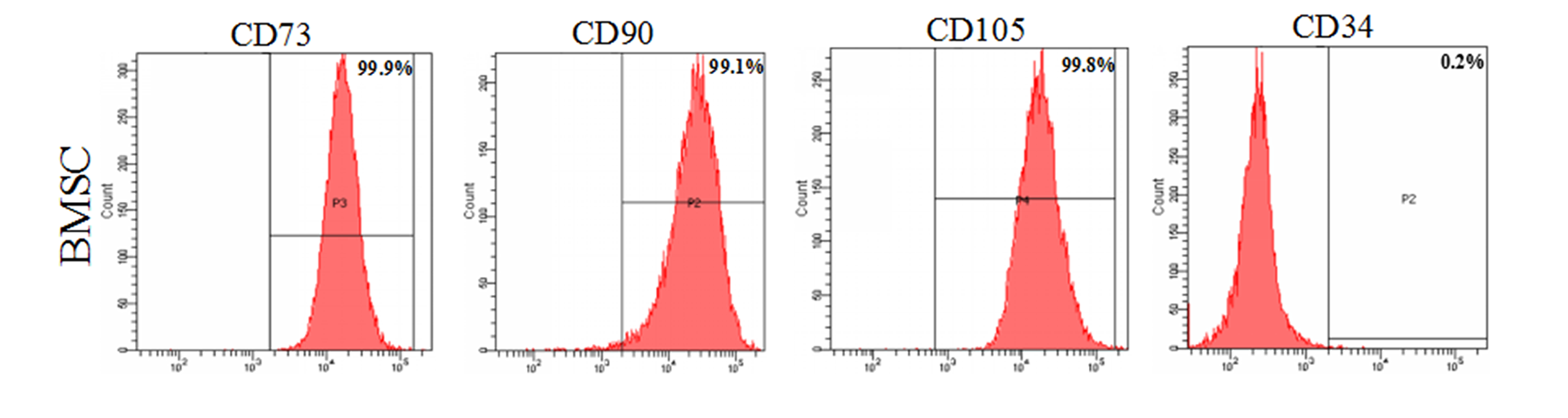

Supplement: Supplementary file 1 — Fig S1 [file JCMM-24-11837-s001.tif]
